# Context-Dependent Associations of PROGINS Variants with Progesterone Receptor Signaling and Biological Features in Diffuse Gliomas

**DOI:** 10.3390/cancers18172835

**Published:** 2026-09-01

**Authors:** Ozan Başkurt, Özlem Kurnaz Gömleksiz, Ege Coşkun, Caner Ünlüer, Merve Nur Aksakal, Bahti Raihanatou Kadijatou, Mahmut Özden, Melih Bozkurt

**Affiliations:** 1 Department of Neurosurgery, Acıbadem Maslak Hospital, Istanbul 34457, Turkey; 2 Department of Medical Biology, Faculty of Medicine, Altınbaş University, Istanbul 34217, Turkey; ozlem.kurnaz@altinbas.edu.tr; 3 Central Research Laboratory (AU-MERLAB), Altınbaş University, Istanbul 34217, Turkey; 4 Department of Medical Biology and Genetics, Faculty of Medicine, Mersin University, Mersin 33343, Turkey; 5 Department of Biomedical Sciences, Altınbaş University, Istanbul 34217, Turkey; 6 Department of Neurosurgery, Istanbul Training and Research Hospital, Istanbul 34098, Turkey

**Keywords:** diffuse glioma, progesterone receptor, *PGR* variant, PROGINS, V660L, H770H, Ki-67, p53

## Abstract

**Background:** Progesterone signaling has been implicated in diffuse glioma biology, but the factors contributing to variability in progesterone receptor (*PGR*) activity remain incompletely understood. We investigated whether PROGINS-related *PGR* variants detected in tumor-derived DNA were associated with *PGR* expression and selected biological features of diffuse gliomas. **Methods:** This retrospective translational study included 66 patients with histopathologically confirmed diffuse gliomas. Three PROGINS-associated *PGR* variants—the Alu insertion, V660L (rs1042838), and H770H (rs1042839)—were analyzed using DNA extracted from fresh-frozen tumor tissue. Relative *PGR* gene expression, tissue PGR protein concentrations, Ki-67 labeling index, and p53 immunoreactivity were evaluated together with IDH status, ATRX expression, and 1p/19q co-deletion. Separate multivariable regression models assessed variant-specific associations and prespecified variant × sex and variant × tumor grade interactions. Multiple testing within the reported multivariable models was addressed using the Benjamini–Hochberg false discovery rate procedure. **Results:** Relative *PGR* gene expression was lower in high-grade than in low-grade gliomas (*p* = 0.039), whereas tissue PGR protein concentrations did not differ significantly according to grade. In multivariable analyses, V660L L-allele carriage was associated with higher *PGR* expression and lower Ki-67 labeling indices, H770H G-allele carriage was associated with lower *PGR* expression and higher p53 immunoreactivity, and Alu insertion carrier status was associated with lower *PGR* expression. Significant variant × sex interactions were identified for several outcomes, and an H770H × tumor grade interaction was observed for *PGR* expression. All nominally significant associations reported in the multivariable models remained significant after false discovery rate correction. **Conclusions:** PROGINS-related *PGR* variants detected in tumor-derived DNA are associated with selected biological features of diffuse gliomas, with several associations differing according to biological sex and, for selected outcomes, tumor grade. These findings support a context-dependent relationship between *PGR* variant status and glioma phenotype but do not establish functional causality or clinical biomarker utility. Independent functional and longitudinal studies are required to determine the biological and potential clinical relevance of these associations.

## Simple Summary

Diffuse gliomas are biologically diverse brain tumors, and progesterone signaling may contribute to this heterogeneity. In this study, we examined whether three progesterone receptor (*PGR*) gene variants associated with the PROGINS haplotype were related to receptor expression and selected biological characteristics of diffuse gliomas. The variants were analyzed in tumor-derived DNA from 66 patients and evaluated together with *PGR* expression, PGR protein concentration, Ki-67 labeling index, p53 immunoreactivity, molecular tumor characteristics, biological sex, and tumor grade. Several associations differed according to sex and, for selected outcomes, tumor grade, and the principal multivariable findings remained significant after correction for multiple testing. These results suggest that the relationship between *PGR* variant status and glioma phenotype is context dependent. However, the findings are observational and do not establish a causal mechanism or clinical biomarker value. Further functional and outcome-based studies are required.

## 1. Introduction

Diffuse gliomas are biologically heterogeneous primary tumors of the central nervous system and remain associated with substantial morbidity and mortality despite advances in surgery, radiotherapy, and systemic treatment [1]. The incorporation of molecular alterations such as isocitrate dehydrogenase (IDH) mutation, alpha thalassemia/mental retardation syndrome X-linked (ATRX) loss, and 1p/19q co-deletion into the 2021 World Health Organization (WHO) classification has transformed glioma diagnosis, prognostic stratification, and therapeutic decision-making [2,3,4]. Nevertheless, considerable biological variability persists among molecularly defined glioma entities, suggesting that additional regulatory pathways may contribute to tumor phenotype.

Among these pathways, steroid hormone signaling has attracted increasing attention because of its context-dependent associations with proliferation, invasion, apoptosis, and therapeutic response [5,6,7,8]. Progesterone, which functions both as a systemic steroid hormone and a neurosteroid synthesized within the central nervous system, exhibits complex effects in experimental glioma models [9,10]. Lower progesterone concentrations have been associated with increased tumor-cell proliferation, migration, and invasion, whereas higher concentrations have been linked to growth inhibition and apoptosis [11,12,13,14]. These observations suggest that progesterone activity may depend not only on hormone concentration but also on the cellular and molecular context in which receptor signaling occurs.

The biological effects of progesterone are mediated in part by nuclear progesterone receptors (PGRs), ligand-activated transcription factors encoded by the *PGR* gene on chromosome 11q22–23 [15,16,17]. The two major isoforms, PGR-A and PGR-B, differ in their transcriptional activities, and variations in receptor abundance, isoform balance, ligand responsiveness, and co-regulator interactions may influence downstream signaling. *PGR* expression has been demonstrated in gliomas, although its relationship with tumor biological characteristics remains inconsistent [13]. Genetic variation within *PGR* may therefore represent an additional factor associated with heterogeneity in progesterone receptor signaling.

The PROGINS haplotype comprises an Alu insertion in intron G, the V660L missense variant (rs1042838) in exon 4, and the H770H synonymous variant (rs1042839) in exon 5 [18,19,20]. These variants have been reported to occur in linkage disequilibrium and have been investigated in relation to PGR expression and function [20,21,22,23,24]. Functional studies have suggested that PROGINS-associated variants may influence receptor transcriptional activity and progesterone responsiveness, although their biological consequences appear to be context dependent [18,19,20]. While these variants have been investigated in several hormone-responsive malignancies, their relationships with *PGR* expression and biological characteristics in diffuse gliomas remain poorly characterized [21,22,24].

In the present study, we investigated the associations of the three PROGINS-related *PGR* variants detected in tumor-derived DNA with *PGR* gene expression, tissue PGR protein concentrations, Ki-67 labeling index, and p53 immunoreactivity in diffuse gliomas. These relationships were evaluated in the context of IDH status, ATRX expression, 1p/19q co-deletion, biological sex, and tumor grade. Because the three PROGINS-related variants are components of the PROGINS haplotype but represent structurally distinct genetic alterations, variant-specific associations were evaluated separately. Our objective was to characterize associations between *PGR* variant status and selected tumor biological features rather than to establish causality or clinical biomarker utility. We hypothesized that these associations would vary according to the biological context of individual tumors.

## 2. Materials and Methods

### 2.1. Study Population and Ethical Approval

This retrospective translational study included adult patients who underwent microsurgical resection for histopathologically confirmed diffuse gliomas at Memorial Bahçelievler Hospital between December 2022 and December 2024. Eligible patients had sufficient tumor tissue available for the required histopathological and molecular analyses. Pediatric tumors, recurrent gliomas, and non-diffuse glioma histologies were excluded. The final analytical cohort comprised 66 patients.

The initial study protocol also included peripheral blood samples from healthy volunteers for exploratory genotype and allele frequency comparisons. However, because the present analysis focused specifically on associations between tumor-derived *PGR* variant status and biological features of diffuse gliomas, healthy controls were not included in the final analytical cohort.

The study was conducted in accordance with the Declaration of Helsinki and was approved by the Memorial Hospital Ethics Committee (Approval No. 56; 26 September 2022). Written informed consent for surgery and the use of anonymized clinical and pathological data for research purposes was obtained from all participants. Molecular analyses were performed at the Altınbaş University Central Research Laboratory (AU-MERLAB), Istanbul, Turkiye.

### 2.2. Neuropathological and Molecular Classification

All tumor specimens underwent neuropathological review, and integrated diagnoses were established according to the 2021 WHO Classification of Tumors of the Central Nervous System [23]. For selected analyses, tumors were additionally categorized as low-grade (WHO grade 2) or high-grade (WHO grades 3–4); this grouping was used as an analytical variable and did not replace the integrated WHO diagnosis.

IDH1 R132H immunohistochemistry was performed routinely. In patients younger than 50 years with negative IDH1 R132H immunostaining, additional molecular testing for *IDH1* and *IDH2* hotspot mutations was performed. IDH-mutant tumors underwent 1p/19q co-deletion analysis by fluorescence in situ hybridization (FISH). Tumors harboring an IDH mutation and 1p/19q co-deletion were classified as oligodendroglioma, IDH-mutant and 1p/19q-codeleted, whereas IDH-mutant tumors without 1p/19q co-deletion were classified as astrocytoma, IDH-mutant.

IDH-wildtype diffuse astrocytic tumors were classified using integrated histopathological and available molecular findings. Tumors with histological WHO grade 4 features, including necrosis and/or microvascular proliferation, were classified as glioblastoma, IDH-wildtype. *TERT* promoter mutation, *EGFR* amplification, and combined chromosome 7 gain/chromosome 10 loss were not systematically assessed in all cases and were therefore not uniformly available for classification.

Immunohistochemistry was performed using a Dako Omnis automated platform (Agilent Technologies, Santa Clara, CA, USA). Ki-67 (MIB-1; Dako, Santa Clara, CA, USA) and p53 (DO-7; Dako) labeling indices were assessed on digitally scanned slides (Aperio AT2; Leica Biosystems, Wetzlar, Germany) using Sectra IDS7 software, version 26.2.12.8350 (Sectra AB, Linköping, Sweden). At least 2000 tumor nuclei were evaluated per case, and labeling indices were expressed as the percentage of positively stained nuclei. IDH1 R132H (GeneAb, Geel, Belgium) and ATRX (Medaysis, Reno, NV, USA) immunostaining was performed according to the manufacturers’ protocols. ATRX expression was classified as retained or lost; loss was defined as absent nuclear staining in tumor cells with preserved staining in non-neoplastic internal control cells.

### 2.3. Tumor Tissue Processing and Molecular Analyses

Representative tumor tissue was snap-frozen in liquid nitrogen and stored at −80 °C until analysis. Before molecular testing, hematoxylin–eosin-stained sections were reviewed to confirm tumor presence and tissue adequacy and to assess necrosis. Representative tumor-containing areas were selected for molecular analyses; for FISH, these areas were specifically marked on digitally scanned slides.

Fresh-frozen tumor samples were homogenized, and DNA, total RNA, and protein were extracted from approximately 20–40 mg of tissue using the Nucleogene DNA, Total RNA, and Protein Extraction Kit (Nucleogene, Türkiye; Cat. No. NGE011) according to the manufacturer’s protocol. DNA and RNA concentrations were measured using a Nano-300 Micro-spectrophotometer (Hangzhou Allsheng Instruments Co., Ltd., Hangzhou, China).

*IDH1* codon 132 and *IDH2* codons 140 and 172 hotspot mutations were analyzed using real-time polymerase chain reaction (PNAClamp™ Mutation Detection Kit; PNAC-4001, PNAC-5101, Panagene Inc., Daejeon, Republic of Korea). Chromosomal 1p/19q co-deletion was assessed by FISH using the ZytoVision Glioma 1p/19q Probe Set (ZytoVision GmbH, Bremerhaven, Germany; Cat. No. Z-2271-20), with at least 100 representative tumor nuclei evaluated per case. Formalin-fixed paraffin-embedded tissue was used for routine diagnostic molecular procedures when required.

Because *PGR* gene expression and protein concentrations were measured in bulk tumor tissue, contributions from non-neoplastic neural, inflammatory, vascular, or stromal components could not be completely excluded.

### 2.4. PGR Variant Analysis

Three PROGINS-associated *PGR* variants were investigated: the Alu insertion in intron G, V660L (rs1042838), and H770H (rs1042839). In the glioma cohort, genotyping was performed using DNA extracted from fresh-frozen tumor tissue.

The Alu insertion was genotyped by conventional PCR using the forward primer 5′-ATACGGTATCCATGACATGAG-3′ and reverse primer 5′-AAGTATTTTCTTGCTAAATGTCTG-3′. Amplification was performed in a 20 µL reaction containing 15 ng DNA, 10 pmol of each primer, 200 nM dNTPs, 1× Taq polymerase buffer, 1 U Taq polymerase, and 1.5 mM MgCl_2_. Cycling conditions consisted of initial denaturation at 94 °C for 10 min; 35 cycles of 94 °C for 20 s, 51 °C for 20 s, and 72 °C for 60 s; and final extension at 72 °C for 10 min. PCR products representing the non-insertion (65 bp) and insertion (385 bp) alleles were separated on 2% agarose gels.

V660L and H770H were genotyped using TaqMan^®^ SNP Genotyping Assays (Life Technologies, Carlsbad, CA, USA; Cat. No. 4351379) according to the manufacturer’s protocol, with endpoint genotyping performed using LightCycler 96 System (Roche Diagnostics GmbH, Mannheim, Germany; software V1.1).

For association analyses, dominant genetic models were used. T1T2/T2T2 genotypes were classified as Alu insertion carriers, CA/AA as V660L L-allele carriers, and AG/GG as H770H G-allele carriers, with T1T1, CC, and AA serving as the respective reference groups.

Because patient genotyping was performed using tumor-derived DNA without systematically paired constitutional DNA, the detected variants were not considered confirmed germline variants and are referred to as *PGR* variants or tumor-derived *PGR* variant status throughout the manuscript.

### 2.5. PGR Gene Expression and Protein Quantification

Total RNA from fresh-frozen tumor tissue was reverse-transcribed into complementary DNA using a commercial cDNA synthesis kit (Nucleogene, Istanbul, Türkiye; Cat. No. NG064). Relative *PGR* gene expression was quantified by real-time quantitative PCR using the LightCycler 96 System (Roche Diagnostics GmbH, Mannheim, Germany), normalized to *GAPDH*, and calculated using the 2^−ΔΔCt^ method.

Tissue PGR protein concentrations were quantified using a commercially available enzyme-linked immunosorbent assay (ELISA Cat. No. E1084Hu; BT Lab, Shanghai, China) according to the manufacturer’s instructions, with absorbance measured at 450 nm.

### 2.6. Statistical Analysis

Statistical analyses were performed using IBM SPSS Statistics version 24.0 (IBM Corp., Armonk, NY, USA). Data distribution was assessed using the Shapiro–Wilk test. Continuous variables are presented as mean ± standard deviation or median (interquartile range), as appropriate, and categorical variables are presented as frequencies and percentages.

Normally distributed continuous variables were compared using the independent-samples *t* test, whereas non-normally distributed variables were analyzed using the Mann–Whitney *U* or Kruskal–Wallis *H* test, as appropriate. Categorical variables were compared using Pearson’s chi-square or Fisher’s exact test. Exploratory comparisons were also performed across the integrated WHO entities represented in the cohort: glioblastoma, IDH-wildtype; astrocytoma, IDH-mutant; and oligodendroglioma, IDH-mutant and 1p/19q-codeleted. To address the potential biological heterogeneity introduced by combining WHO grade 3 and grade 4 tumors, a post hoc sensitivity analysis was performed.

Separate multivariable linear regression models were constructed for each *PGR* variant rather than entering all three PROGINS-associated variants simultaneously into the same model. *PGR* gene expression, Ki-67 labeling index, and p53 immunoreactivity were log10-transformed before regression analysis. Models included variant carrier status, age, biological sex, tumor grade, IDH status, ATRX expression status, and 1p/19q co-deletion status. Prespecified variant × sex and variant × tumor grade interaction terms were evaluated. Regression coefficients (β) are reported with 95% confidence intervals. Model assumptions were assessed using residual diagnostics, and multicollinearity was evaluated using variance inflation factors.

To account for multiple testing within the prespecified multivariable models, nominal *p* values for genotype main effects and interaction terms were adjusted using the Benjamini–Hochberg false discovery rate (FDR) procedure. Both nominal *p* values and FDR-adjusted *q* values are reported, with *q* < 0.05 considered statistically significant.

## 3. Results

### 3.1. Patient Characteristics and Tumor Profile

A total of 66 patients with histopathologically confirmed diffuse gliomas were included in the final analytical cohort. According to the 2021 WHO classification, 19 tumors (28.8%) were classified as low-grade (WHO grade 2) and 47 (71.2%) as high-grade (WHO grades 3–4). Integrated diagnoses included 34 glioblastomas (51.5%), 19 astrocytomas (28.8%), and 13 oligodendrogliomas (19.7%). Selected demographic and biological characteristics of the study population are summarized in Table 1.

Patients with high-grade gliomas were significantly older than those with low-grade tumors (*p* = 0.007). The Ki-67 labeling index was significantly higher in high-grade gliomas (*p* < 0.001), whereas p53 immunoreactivity did not differ significantly between grade groups (*p* = 0.118). IDH-wildtype tumors were more frequent among high-grade gliomas (*p* = 0.007). Similarly, retained ATRX expression predominated among high-grade gliomas, whereas ATRX loss was more frequent among low-grade tumors (*p* = 0.033).

Relative *PGR* gene expression was significantly lower in high-grade than in low-grade gliomas (*p* = 0.039), whereas tissue PGR protein concentrations did not differ significantly according to tumor grade (*p* > 0.05). Among low-grade tumors, 1p/19q-codeleted gliomas showed lower *PGR* gene expression than non-codeleted tumors (*p* = 0.020); this association was also observed in the exploratory analysis restricted to male patients (*p* = 0.015). Among females with high-grade gliomas, IDH-mutant tumors showed higher tissue PGR protein concentrations than IDH-wildtype tumors (*p* = 0.010).

In a post hoc sensitivity analysis separating WHO grade 3 (*n* = 10) and grade 4 (*n* = 37) tumors, no significant differences were observed in *PGR* gene expression (*p* = 0.150), PGR tissue protein concentration (*p* = 0.866), Ki-67 labeling index (*p* = 0.443), or p53 immunoreactivity (*p* = 0.769). Thus, although grade 3 and grade 4 tumors are histopathologically distinct, separation of these groups did not identify significant differences in the biological variables evaluated in the present study.

Because the three integrated WHO tumor entities were represented by unequal and relatively small subgroups, particularly after further stratification by genotype and sex, additional fully adjusted genotype-by-tumor-entity interaction analyses were not performed. Tumor grade was therefore retained as a prespecified analytical context variable and was not interpreted as a substitute for integrated molecular diagnosis.

### 3.2. Genotype and Allele Distributions of PGR Variants

Genotype and allele distributions of the three PROGINS-associated *PGR* variants are summarized in Table 2. No statistically significant differences in genotype or allele frequencies were observed between low- and high-grade gliomas for the Alu insertion, V660L (rs1042838), or H770H (rs1042839) variants (all nominal *p* > 0.05).

For subsequent association analyses, dominant carrier models were used. Alu insertion carriers were defined as T1T2/T2T2, V660L L-allele carriers as CA/AA, and H770H G-allele carriers as AG/GG. These definitions were used consistently in the exploratory subgroup analyses and multivariable models.

### 3.3. Associations Between PGR Variants and Tumor Biological Features

The subgroup comparisons presented below were exploratory and were used to characterize patterns subsequently evaluated in multivariable interaction models. Given the limited sample sizes within genotype-by-sex and genotype-by-grade strata, these unadjusted subgroup findings should not be interpreted as independent confirmatory associations.

Alu insertion: No significant associations between Alu insertion carrier status and the evaluated tumor biological markers were identified in the overall cohort (nominal *p* > 0.05). However, exploratory sex-stratified analyses suggested divergent patterns of *PGR* gene expression in high-grade gliomas. Among males with high-grade tumors, *PGR* gene expression was higher in non-carriers (T1T1) than in Alu insertion carriers (T1T2/T2T2) (*p* = 0.002). Conversely, among females with high-grade tumors, Alu insertion carriers showed higher *PGR* gene expression than non-carriers (*p* = 0.002). No significant associations were observed in low-grade gliomas.

V660L: V660L L-allele carriage was associated with higher tissue PGR protein concentrations than the CC reference genotype in the overall cohort (*p* = 0.015), with a stronger difference observed in the exploratory low-grade subgroup (*p* < 0.001). In contrast, the CC genotype was associated with higher Ki-67 labeling indices than L-allele carriage (*p* = 0.022).

A borderline association was observed between V660L genotype and mutant-pattern p53 immunostaining (*p* = 0.050). In an exploratory analysis restricted to females with high-grade gliomas, the CC genotype was associated with higher p53 immunoreactivity than L-allele carriage (*p* < 0.001). No significant associations were identified with age, IDH status, ATRX expression status, or relative *PGR* gene expression.

H770H: H770H G-allele carriage was associated with higher p53 immunoreactivity than the AA reference genotype (*p* = 0.029) and with a higher frequency of mutant-pattern p53 immunostaining (*p* = 0.050). The difference in p53 immunoreactivity was also observed in the exploratory low-grade subgroup (*p* = 0.005).

Additional sex- and grade-stratified exploratory analyses suggested lower *PGR* gene expression among G-allele carriers in males with high-grade gliomas (*p* = 0.003) and higher p53 immunoreactivity among G-allele carriers in females with high-grade gliomas (*p* = 0.043). No significant associations were identified with the Ki-67 labeling index, tissue PGR protein concentration, IDH status, or ATRX expression status.

Because these subgroup analyses involved multiple comparisons and small strata, they are considered hypothesis-generating. The principal inferential results are therefore based on the prespecified multivariable models described below.

### 3.4. Multivariable Regression and Interaction Analyses

Separate multivariable linear regression models were constructed for each *PGR* variant to evaluate associations with tumor biological features after adjustment for age, biological sex, tumor grade, IDH status, ATRX expression status, and 1p/19q co-deletion status. The reported main-effect and interaction *p* values were additionally adjusted using the Benjamini–Hochberg FDR procedure (Table 3).

For V660L, L-allele carriage was independently associated with higher relative *PGR* gene expression (β = +0.214, 95% CI 0.081–0.347; *p* = 0.002; *q* = 0.008). A significant genotype × sex interaction was also observed for *PGR* expression (β = +0.131, 95% CI 0.012–0.250; *p* = 0.031; *q* = 0.034). L-allele carriage was inversely associated with Ki-67 labeling index (β = −0.168, 95% CI −0.289 to −0.047; *p* = 0.007; *q* = 0.014), with a significant genotype × sex interaction (β = −0.141, 95% CI −0.263 to −0.019; *p* = 0.024; *q* = 0.029).

For H770H, G-allele carriage was independently associated with lower relative *PGR* gene expression (β = −0.198, 95% CI −0.326 to −0.070; *p* = 0.003; *q* = 0.009). Significant genotype × sex (β = +0.173, 95% CI 0.052–0.294; *p* = 0.006; *q* = 0.014) and genotype × tumor grade interactions (β = −0.149, 95% CI −0.274 to −0.024; *p* = 0.019; *q* = 0.025) were identified. G-allele carriage was also associated with higher p53 immunoreactivity (β = +0.226, 95% CI 0.089–0.363; *p* = 0.001; *q* = 0.008), with a significant genotype × sex interaction (β = +0.158, 95% CI 0.029–0.287; *p* = 0.017; *q* = 0.025).

For the Alu insertion, carrier status was associated with lower relative *PGR* gene expression (β = −0.162, 95% CI −0.296 to −0.028; *p* = 0.018; *q* = 0.025). A significant genotype × sex interaction was observed (β = +0.241, 95% CI 0.094–0.388; *p* = 0.002; *q* = 0.008), whereas the genotype × tumor grade interaction was not statistically significant (β = −0.113, 95% CI −0.232 to 0.006; *p* = 0.062; *q* = 0.062).

**Figure 1:**
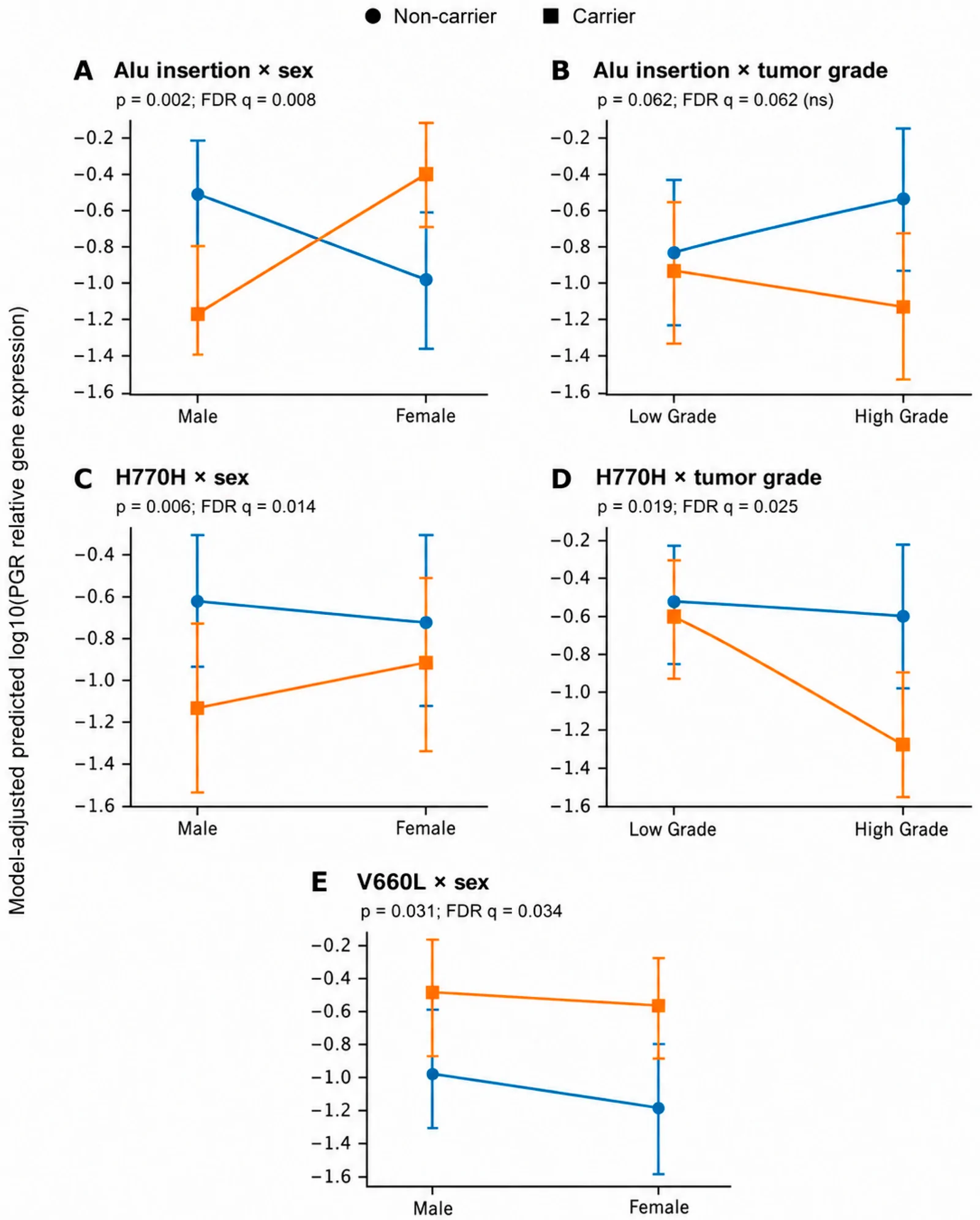
Adjusted interaction plots for *PGR* relative gene expression according to PROGINS-associated variant status. Model-adjusted predictions of log10-transformed *PGR* relative gene expression are shown with 95% confidence intervals for carriers and non-carriers according to biological sex and tumor grade. Panels represent (**A**) Alu insertion × sex, (**B**) Alu insertion × tumor grade, (**C**) H770H × sex, (**D**) H770H × tumor grade, and (**E**) V660L × sex. Predictions were derived from separate multivariable linear regression models adjusted for age, biological sex, tumor grade, IDH status, ATRX expression status, and 1p/19q co-deletion. Nominal interaction *p* values and Benjamini–Hochberg FDR-adjusted *q* values are shown. Error bars represent model-based 95% confidence intervals. ns, not significant.

**Figure 2:**
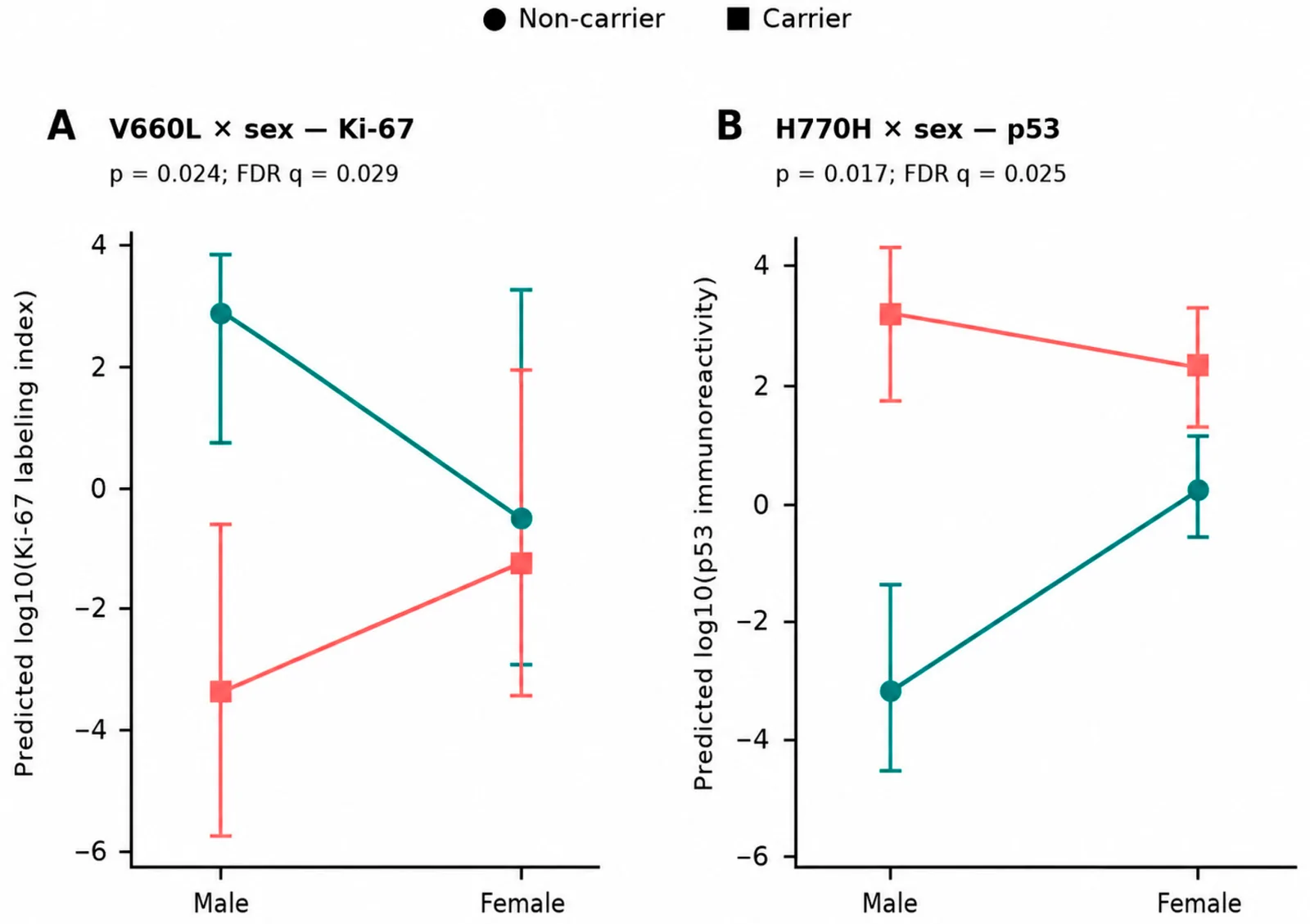
Adjusted interaction plots for selected tumor biological features according to PROGINS-associated variant status. (**A**) Model-adjusted predictions of log10-transformed Ki-67 labeling index according to V660L L-allele carrier status and biological sex. (**B**) Model-adjusted predictions of log10-transformed p53 immunoreactivity according to H770H G-allele carrier status and biological sex. Predictions were derived from separate multivariable regression models adjusted for age, biological sex, tumor grade, IDH status, ATRX expression status, and 1p/19q co-deletion. Nominal interaction *p* values and Benjamini–Hochberg FDR-adjusted *q* values are shown. Error bars represent model-based 95% confidence intervals.

Importantly, all associations that were nominally significant in the reported multivariable models remained significant after FDR correction. Overall, these findings indicate that the associations between PROGINS-related *PGR* variant status and selected tumor biological features differ according to biological sex and, for some outcomes, tumor grade, supporting a context-dependent pattern of association without establishing a causal effect on progesterone receptor signaling or tumor behavior (Figure 1 and Figure 2).

## 4. Discussion

The present study identified associations between PROGINS-related *PGR* variants detected in tumor-derived DNA and selected biological characteristics of diffuse gliomas. The principal findings were that *PGR* gene expression was lower in high-grade than in low-grade tumors, while tissue PGR protein concentrations did not differ significantly according to grade, and that the Alu insertion, V660L, and H770H variants showed distinct associations with *PGR* expression, Ki-67 labeling index, and p53 immunoreactivity. Several of these associations differed according to biological sex and, for H770H, tumor grade. Importantly, the significant main effects and interaction terms reported in the multivariable models remained significant after FDR correction. These findings support a heterogeneous, context-dependent relationship between *PGR* variant status and glioma biological features, but they do not establish a causal effect of these variants on progesterone receptor function or tumor behavior.

One notable finding was the lower *PGR* gene expression observed in high-grade gliomas. Previous studies have reported heterogeneous patterns of progesterone receptor expression across glioma grades, reflecting the complex role of steroid signaling in these tumors [13,25,26]. In the present cohort, lower transcript levels in high-grade tumors were not accompanied by a corresponding difference in tissue PGR protein concentrations. The relationship between mRNA abundance and protein concentration is influenced by multiple post-transcriptional and translational processes, including mRNA stability, translation efficiency, and protein turnover [26,27]. The observed discordance therefore suggests that transcript abundance and tissue protein concentration capture different aspects of PGR biology. However, because receptor turnover, ligand binding, transcriptional activity, and PGR-A/PGR-B isoform balance were not directly assessed, the mechanisms underlying this finding cannot be determined from the present data [28].

The relationship between PGR biology and established glioma molecular characteristics was also heterogeneous. Lower *PGR* expression was observed in 1p/19q-codeleted low-grade tumors, whereas IDH status and ATRX expression patterns differed according to tumor grade, as expected within the integrated molecular landscape of diffuse gliomas. These observations should not be interpreted as evidence that progesterone signaling constitutes an additional classification marker. Rather, they indicate that PGR-related biological features may vary across molecularly distinct tumors. This distinction is particularly important because glioblastoma, IDH-mutant astrocytoma, and IDH-mutant and 1p/19q-codeleted oligodendroglioma represent biologically distinct WHO entities [29]. In the present study, tumor grade was used as a prespecified analytical context variable rather than as a surrogate for integrated molecular diagnosis.

Although WHO grade 3 and grade 4 tumors differ histopathologically, particularly with respect to necrosis and microvascular proliferation, a post hoc sensitivity analysis did not identify significant differences between these groups in *PGR* gene expression, PGR tissue protein concentration, Ki-67 labeling index, or p53 immunoreactivity. This finding suggests that the prespecified grouping of grade 3 and grade 4 tumors as a high-grade analytical category was unlikely to be the primary explanation for the principal observed associations. The sample sizes within individual WHO entities and genotype strata were insufficient to support stable fully adjusted entity-specific interaction models, and this heterogeneity should be considered when interpreting the pooled analyses.

The variant-specific findings further suggest that associations with PGR-related tumor characteristics are not uniform across the three PROGINS components. For the Alu insertion, the principal finding was a genotype × sex interaction for *PGR* gene expression. Exploratory subgroup analyses suggested opposite expression patterns in males and females with high-grade tumors, whereas the genotype × grade interaction was not statistically significant. Previous functional studies have suggested that the PROGINS-associated Alu insertion may affect transcriptional regulation and transcript stability [18,19,20]. Our findings are compatible with the possibility that the association between Alu insertion status and receptor expression varies according to biological context; however, they do not demonstrate altered transcription, receptor activity, or hormone responsiveness. The sex-related pattern is therefore best regarded as a hypothesis-generating observation requiring functional validation.

V660L showed a different pattern. L-allele carriage was independently associated with higher *PGR* gene expression and lower Ki-67 labeling indices, and significant genotype × sex interactions were identified for both outcomes. V660L is a missense variant and has previously been investigated in relation to PGR structure and transcriptional function [24]. Nevertheless, the present study did not directly assess receptor conformation, ligand affinity, nuclear localization, dimerization, or downstream transcriptional signaling. Consequently, the observed associations with receptor expression and proliferative activity should not be interpreted as evidence that V660L directly suppresses proliferation or alters receptor function. Rather, they identify an adjusted statistical association within this cohort that warrants mechanistic investigation [19,30].

H770H was independently associated with lower *PGR* gene expression and higher p53 immunoreactivity, with significant genotype × sex and genotype × grade interactions for selected outcomes. Although H770H is a synonymous variant, synonymous substitutions can potentially be associated with differences in RNA processing, transcript stability, or translational regulation; however, no such mechanism was examined directly in this study [19,31]. The association with p53 immunoreactivity likewise does not establish a functional interaction between PGR and the p53 pathway [25]. The current findings therefore indicate an association between H770H variant status and selected tumor biological markers but cannot determine whether H770H itself is functionally responsible or whether the observed relationship reflects linkage with other genetic variation.

Biological sex emerged as a recurrent effect modifier in the multivariable analyses [13,14]. Genotype × sex interactions were identified for *PGR* expression with all three variants and for Ki-67 with V660L and p53 immunoreactivity with H770H. Sex-related differences in steroid hormone exposure, receptor regulation, and transcriptional co-regulator activity provide a biologically plausible framework for such observations [14,32,33,34]. However, the present study did not include circulating progesterone concentrations, menopausal status, menstrual-cycle phase, exogenous hormone exposure, or use of hormone-modulating medications. Accordingly, biological sex should not be interpreted as a direct surrogate for hormonal exposure. The observed interactions identify sex as a relevant stratification variable for future investigations rather than establishing a specific endocrine mechanism.

The statistical findings also require appropriate qualification. The cohort was relatively small, particularly after stratification by genotype, sex, and tumor grade, and the unadjusted subgroup analyses were intended to be exploratory. For this reason, the main inferential emphasis was placed on the prespecified multivariable models. Benjamini–Hochberg FDR correction was applied to the reported genotype main effects and interaction terms, and all nominally significant associations in these models remained significant after correction. This increases confidence that the principal multivariable findings are not explained solely by the number of reported tests. Nevertheless, the exploratory subgroup comparisons remain susceptible to type I error and should be interpreted as hypothesis-generating rather than independently confirmatory.

Several additional limitations should be acknowledged. First, this was a retrospective, single-center study with 66 patients, limiting statistical power for analyses within individual WHO-defined tumor entities and rare genotype strata. Second, *PGR* genotyping in the glioma cohort was performed using tumor-derived DNA without systematically paired constitutional DNA; consequently, the detected variants cannot be confirmed as germline in individual patients. Cohort-specific pairwise linkage disequilibrium estimates were also not available for the final analytical dataset, limiting direct assessment of the haplotype structure within this cohort. Third, *PGR* gene expression and protein concentrations were measured in bulk tumor tissue. Although representative tumor tissue was selected histologically, contributions from non-neoplastic neural, inflammatory, vascular, or stromal cells cannot be completely excluded. Fourth, functional experiments assessing PGR transcriptional activity, ligand responsiveness, downstream signaling, or PGR-A/PGR-B isoform balance were not performed. Fifth, hormonal exposure variables, including circulating progesterone concentrations, menopausal status, menstrual-cycle phase, exogenous hormone use, and hormone-modulating medications, were unavailable. Sixth, *TERT* promoter mutation, *EGFR* amplification, and combined chromosome 7 gain/chromosome 10 loss were not systematically available for all IDH-wildtype tumors. Finally, survival, recurrence, treatment response, and other clinically meaningful outcomes were not evaluated. The present findings therefore cannot support prognostic, predictive, or therapeutic biomarker claims.

Taken together, the results suggest that PROGINS-related *PGR* variant status is associated with selected receptor-related and tumor biological features in diffuse gliomas and that some of these relationships differ according to biological sex and tumor grade. The findings extend the observational evidence linking progesterone receptor biology with glioma heterogeneity but remain hypothesis-generating. Independent cohorts with integrated WHO molecular classification, paired constitutional and tumor DNA, detailed hormonal exposure data, functional receptor studies, and longitudinal clinical outcomes will be required to determine whether these associations reflect biologically meaningful mechanisms or have future clinical relevance.

## 5. Conclusions

PROGINS-related *PGR* variants detected in tumor-derived DNA were associated with selected biological features of diffuse gliomas, including *PGR* expression, Ki-67 labeling index, and p53 immunoreactivity. Several of these associations varied according to biological sex and, for selected outcomes, tumor grade, supporting a context-dependent relationship between *PGR* variant status and glioma phenotype. These observational findings do not establish functional causality or clinical biomarker utility. Independent studies incorporating paired constitutional DNA, functional receptor analyses, detailed hormonal exposure, integrated molecular tumor classification, and longitudinal clinical outcomes are required to determine the biological and potential clinical relevance of these associations.

## Figures and Tables

**Table 1:** Selected clinical and biological characteristics of the study population.

Variable	Low Grade (*n* = 19)	High Grade (*n* = 47)	*p*-Value
**Age (years)**	36.50 ± 17.49	55.53 ± 10.61	0.007 *
**Sex (Male/Female)**	9/10	33/14	0.118
**Ki-67 labeling index (%)**	6.53 ± 5.98	31.28 ± 17.32	<0.001 *
**p53 immunoreactivity (%)**	24.41 ± 25.29	35.29 ± 30.51	0.118
***PGR* relative gene expression**	0.057 ± 0.081	0.019 ± 0.021	0.039 *
**PGR tissue protein concentration (ng/mL)**	2.86 ± 1.78	2.89 ± 1.75	>0.05

Legends: Continuous variables are presented as mean ± standard deviation. Comparisons were performed using the Mann–Whitney U test. Categorical variables were compared using the Pearson chi-square test or Fisher’s exact test when appropriate. * *p* < 0.05, statistically significant. Abbreviations: *PGR*, progesterone receptor.

**Table 2:** Genotype, allele, and carrier definitions of *PGR* variants according to glioma grade.

	Genotype/Allele and Carrier Definition	Low Grade	High Grade	*p*-Value
**Alu insertion/PROGINS**	T1T1/Alu−Alu−, non-carrier	78.6%	73.8%	>0.05
Wild-type allele: T1/Alu−	T1T2/Alu−Alu+, insertion carrier	14.3%	16.7%	
Variant allele: T2/Alu+	T2T2/Alu+Alu+, insertion carrier	7.1%	9.5%	
	T2 allele frequency	10.7%	8.3%	>0.05
**V660L (rs1042838)**	CC/V/V, L-allele non-carrier	75.0%	77.1%	>0.05
Wild-type allele: C = V allele	CA/V/L, L-allele carrier	21.4%	19.0%	
Variant allele: A = L allele	AA/L/L, L-allele carrier	3.6%	3.9%	
	A allele/L allele frequency	14.3%	13.1%	>0.05
**H770H (rs1042839)**	AA, G-allele non-carrier	75.0%	73.8%	>0.05
Wild-type allele: A allele	AG, G-allele carrier	14.3%	19.0%	
Variant allele: G allele	GG, G-allele carrier	10.7%	7.1%	
	G allele frequency	17.9%	16.7%	>0.05

Legends: Genotype and allele frequencies are presented as percentages. For functional association analyses, genotypes were additionally grouped under a dominant carrier model. Alu insertion carriers were defined as T1T2 or T2T2. For V660L (rs1042838), the C allele corresponds to valine (V), whereas the A allele corresponds to leucine (L); CA and AA genotypes were considered L-allele carriers. For H770H (rs1042839), AG and GG genotypes were considered G-allele carriers. Genotype and allele frequencies were compared using the Pearson chi-square test or Fisher’s exact test where appropriate.

**Table 3:** Variant-specific multivariable linear regression models assessing associations between *PGR* variants and tumor biological markers.

Polymorphism	Outcome	Term	β	95% CI	*p*-Value	FDR *q*-Value
**V660L**	log10(*PGR*)	Genotype	+0.214	0.081–0.347	0.002	0.008
		Genotype × Sex	+0.131	0.012–0.250	0.031	0.034
	log10(Ki-67)	Genotype	−0.168	−0.289–−0.047	0.007	0.014
		Genotype × Sex	−0.141	−0.263–−0.019	0.024	0.029
**H770H**	log10(*PGR*)	Genotype	−0.198	−0.326–−0.070	0.003	0.009
		Genotype × Sex	+0.173	0.052–0.294	0.006	0.014
		Genotype × Grade	−0.149	−0.274–−0.024	0.019	0.025
	log10(p53)	Genotype	+0.226	0.089–0.363	0.001	0.008
		Genotype × Sex	+0.158	0.029–0.287	0.017	0.025
**Alu insertion**	log10(*PGR*)	Genotype	−0.162	−0.296–−0.028	0.018	0.025
		Genotype × Sex	+0.241	0.094–0.388	0.002	0.008
		Genotype × Grade	−0.113	−0.232–0.006	0.062	0.062

Legends: Separate multivariable linear regression models were constructed for each PROGINS-associated *PGR* variant. Continuous outcomes were log10-transformed before analysis. Models were adjusted for age, biological sex, tumor grade, IDH status, ATRX expression status, and 1p/19q co-deletion status. Genotypes were analyzed using dominant carrier models. β values represent adjusted regression coefficients and are reported with 95% confidence intervals. Multiple testing across the 12 reported genotype main-effect and interaction tests was controlled using the Benjamini–Hochberg FDR procedure. FDR-adjusted *q* < 0.05 was considered statistically significant. Abbreviations: *PGR*, progesterone receptor; FDR, false discovery rate.

## Data Availability

The datasets generated and/or analyzed during the current study are not publicly available because they contain information that could compromise participant privacy but are available from the corresponding author upon reasonable request, subject to applicable ethical and privacy restrictions.

## Abbreviations

The following abbreviations are used in this manuscript:

PGR	progesterone receptor
WHO	World Health Organization
IDH	isocitrate dehydrogenase
ATRX	alpha thalassemia/mental retardation syndrome X-linked
FISH	fluorescence in situ hybridization
PCR	polymerase chain reaction
CI	confidence interval
FDR	false discovery rate
